# Supplementary figures and images for: Adaptation mechanism of the adult zebrafish respiratory organ to endurance training
Source: PLoS One. 2020 Feb 5;15(2):e0228333. doi: 10.1371/journal.pone.0228333 (PMC7001924; doi:10.1371/journal.pone.0228333)

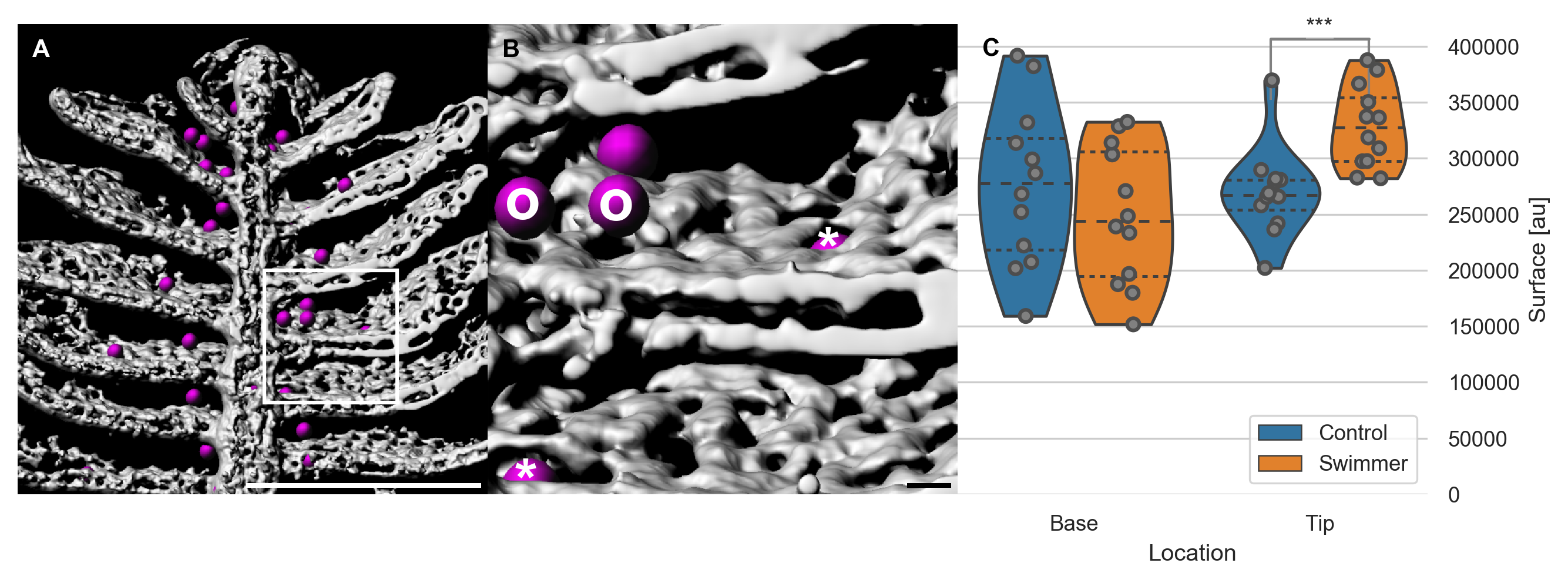

Supplement: S1 Fig — A: Endothelial surface in grey (based on relative eGFP channel intensity), showing the capillary network. Scale bar: 0.1 mm. B: Detailed view of square in A. Examples of BrdU positive endothelial cells (*) and of BrdU-positive cells of other origin (o). Scale bar: 5 μm. C: Plots of the estimated surface at the base and tips (p = 0.00053). (TIF) [file pone.0228333.s001.tif]

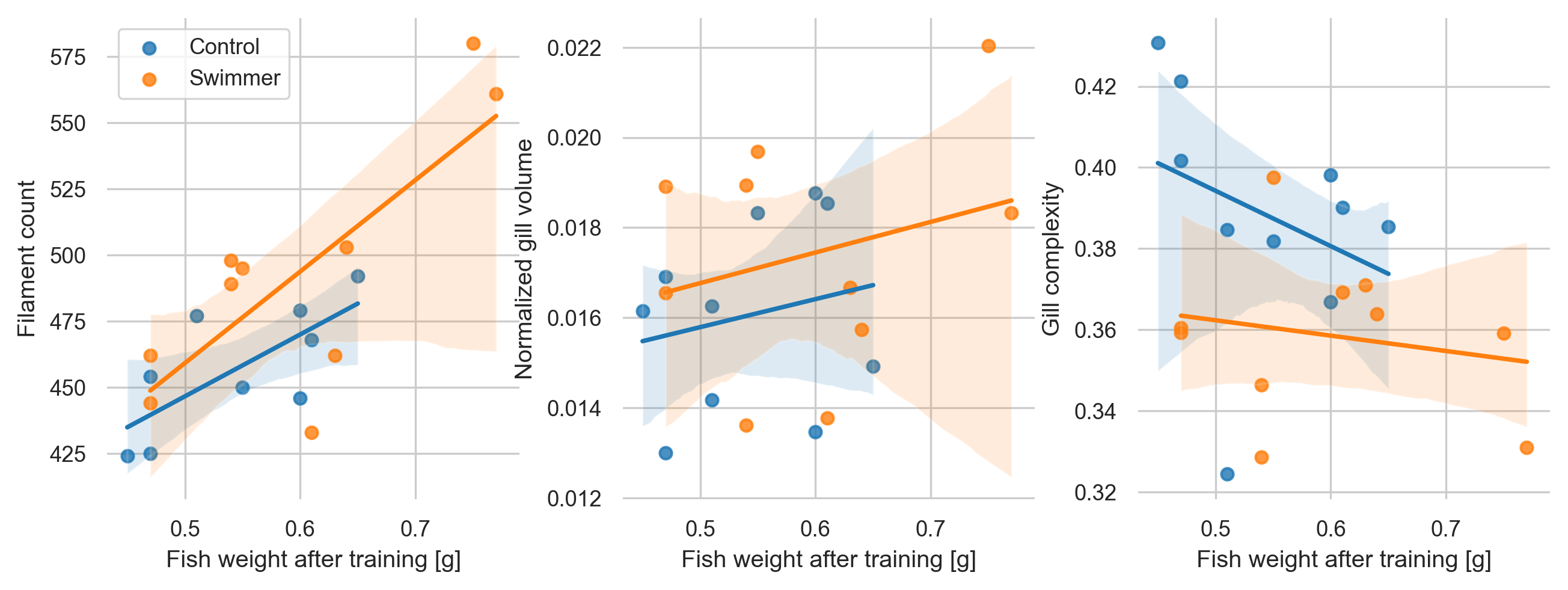

Supplement: S2 Fig — Left: Weight after training to filament count. R2 controls 0.518 (p = 0.029, *). R2 swimmers 0.576 (p = 0.011, *). Middle: Weight after training to normalized) gill volume. R2 controls 0.042 (p = 0.57, n.s.). R2 swimmers 0.071 (p = 0.46, n.s.). Right: Weight after training to gill complexity. R2 controls 0.105 (p = 0.36, n.s.). R2 swimmers 0.039 (p = 0.59, n.s.). The translucent bands mark the 95% confidence interval. (TIF) [file pone.0228333.s002.tif]
